# Indents

**Published:** 1921-02

**Authors:** 


### INDENTS

**☞Brief Articles of Technical or Scientific Value will receive notice and comment. Contributions invited.**

---

THE DENTAL EDUCATIONAL COUNCIL OF AMERICA. The education of the future dentist has been delegated to the colleges of the country and nobly have they fulfilled their mission. Their task has not been an easy one, for the rapid advancement of the profession in scientific thought and professional attainment has placed an ever-changing condition upon the colleges and their teaching equipment that has meant no small burden to them. In spite of this they have, for the most part, valiently carried on their work and we owe much of our present position to their untiring efforts and zealous endeavors. However, the educational conditions of our various institutions was in a very chaotic condition some few years back owing to the difference of educational ideals, and opportunities present in the different colleges. So apparent was this to the examining bodies that they suggested the organization of an advisory body composed of five members from each of the national dental organizations. Therefore the Dental Educational Council of America was formed with a membership of five from the National Examiners' Association, five from the National Faculties' Association, and five from the National Dental Association. The work of this body has been productive of the greatest good, and out of the chaotic conditions of the past has been evolved an educational system that is a unit in regard to the preliminary entrance requirements, the curriculum and the management of the institutions in regard to commercialism.

Several colleges that were not living up to the ideals of the profession have been stimulated to better efforts and are rapidly approaching the highest attainment in educational ideals. The greatest good that the council might have attained has been made impossible by the refusal of one branch of the colleges to affiliate with the Educational

Council, but in spite of that drawback, the work has progressed in such a manner that it has received the approbation of the United States Government, and during the period of the war was made the official medium of the government's activities in dental education. The future progress of education in the dental profession depends upon a central body which shall have the authority to advise, stimulate and correct the educational activities of the various institutions. I know of no better medium for this work than the one now functionating, representing, as it does, the three great bodies in dental education, legislation and execution. I therefore bespeak for the Dental Educotional Council of America the moral and financial support of the National Dental Association.—*Journal N. D. A.*

---

Indiscriminate Tooth Extraction. We now come to a live question, and that is the question of extraction. We have a group of practitioners in this country who call themselves the "one hundred percenters," who extract every tooth in the mouth where the pulp is not vital, whether there is any destruction of the tissues going on around the apex or not. Have you read the work of Collins of the Research Institute? Do you realize that in fifty per cent. of the teeth affected with pyorrhea or with any disease at all the pulps are infected? If you are going to extract every tooth that you think is infected, where are you going to draw the line? We want to begin to think logically; we want to begin to think of many things and to consider carefully this matter of extraction. This fad of extracting every tooth because we think it is infected is absurd.

I have been watching for years these fads that have come over the profession. Take for instance crown work. We have been crowning everything; we have been wild on the subject.

There are one or two things, however, which I wish to review and dwell on particularly. I want you to think about this: if you do your X-ray work properly, if you examine the teeth, and after having proper radiographs of the teeth, if you think a tooth must be extracted, think of several things. Of course, first is the question of the patient's health. Think of the effect of the loss of that tooth and what it means to the patient, and then think if there is not some method that can be used for the preservation of that tooth. No tooth suspected of being infected, in my opinion, should be extracted unless there have been three distinct views of it taken with the X-ray—taken from three different angles. Keep in mind, too, that one of the worst forms of infection we have around the apex is the rarefying form. If we have a condensed area, the chances for retention are very much greater than in the cases where we have an extensive rarefied area. Finally, keep your balance; keep your mind level; do not run after untried things. The profession has chased a great many butterflies in the last thirty years; some men sacrifice their lives running after butterflies. Let us be a thinking profession; a rational profession; let us be a profession that will be an ornament to this country. Let us do things in a rational way. Let us reason together and when we have reached a conclusion, let us have a firm and solid foundation so that we may have a reason for everything we do.—Dr. T. P. Hinman, in *Cosmos* for November, 1920.

---

THE WIRE CLASP VS. THE CAST CLASP. Claiming no originality for the wire clasp, I merely take the liberty of presenting some argument why I think the wire clasp will very quickly eliminate the cast clasp, and make it almost an inexcusable crime to extract any sound or nearly sound teeth in order to make a beautiful set of gold or platinum teeth, etc. A period of years is not necessary to prove to the ordinary individual that if he can make a restoration that affords comfort, service and nearly perfect esthetic effect, without any tooth mutilation, he has found the long-wanted method; and it does not take very much effort to get any price he thinks his time is worth. Therefore I make no apologies when I give eight reasons why the wire clasp should be used in all partial restorations.

1. The *wire* clasp has but a line of contact, because it is round on the tooth surface.

The *cast* clasp, when its fits, covers two-thirds of the tooth surface.

Which clasp is liable to cause disintegration?

2. The *wire* clasp when properly made has a limited motion under stress.

The *cast* clasp has no motion under stress when it is properly made.

Which clasp allows the more natural tooth movement?

3. The *wire* clasp, because of its motion, allows the saddle to receive nearly all the force of mastication.

The *cast* clasp, because of its rigidity, does not allow the saddle to receive any of the force of mastication.

Which clasp puts the least strain on the abutments?

4. The *wire* clasp can be used no matter how badly the abutments are tipped, without losing any strength.

The *cast* clasp can only be used when the teeth are parallel or so nearly parallel that they can be forced apart to get the bridge into position, or else weaken the bridge.

Which clasp will have the greater possibilities?

5. The *wire* clasp because it is round and because it has motion, is practically self-cleansing.

The *cast* clasp because it has a flat surface, and has no motion harbors secretions.

Examine a cast clasp on the inside and see what you can scrape off.

6. The *wire* clasp because of its limited motion is ideal in cantilever restorations.

The *cast* clasp because of its rigidity is never indicated in cantilever restorations.

Which clasp is lessening our troubles?

7. The *wire* clasp being round, and nearly always at the gum line is seen very little.

The *cast* clasp being over nearly all the tooth surface, nothing else can be seen.

Which clasp does the patient prefer?

8. The *wire* clasp is an evil.

The *cast* clasp is an evil.

We are duty bound to always select the lesser evil in everything we do for our patients. I will leave it to the reader to say which is the lesser evil—W. H. Jordan, *Cosmos.*

---

Oral Prophylax. If the white-haired odontologist will prescribe the doctrine of prophylaxis, particularly to his younger patients, and advise his dentist son to benefit by the new régime of oral hygiene, we may sometime approach that long-sought day when a patient will come in to tell us that a brother dentist recently cleaned his teeth correctly and did not find it necessary to put on even a single gold crown.

If we could only get the happy combination! Education from both directions, the profession and the laity, will solve it. Well-educated dentists should know the propaganda of more prevention and less cure and every school child, by government assistance, should be a living working model of this slogan. What our profession needs is less attention to fixed and removable claptraps and more devotion to a good scientific cleaning of the teeth, respect for healthy gums, closer attention to small cavities and instillation of advice for home use, starting them while they are young. Of course, the necessity for legitimate and clean restorations will never cease to exist, since ofttimes unconquerable forces defeat even our most painstaking efforts to prevent or check disease, which is all the more reason for putting our patients on their guard.

A great medical man once said the best means of teaching was to reiterate, reiterate and reiterate.

Therefore with our hands out of our pockets, humanity in mind, and our professional nobility at stake, let us all repeat, as often as we can, more prevention and less cure. Joseph Herbert Kauffman, *Cosmos*.

---

NOVOCAIN AS A SKIN IRRITANT. Never having seen in literature any reference to novocain as a skin irritant, I think my recent experience in this connection may be of interest.

In the winter of 1919 my hands became highly irritated, chapped, cracked, and at times badly swollen, especially the third and fourth fingers of the right, and index finger and thumb of the left hand. In May my face and lips began to itch and became badly swollen. Both my hands and face itched to such an extent that I spent many sleepless nights in nothing short of misery. In July my face broke out with yellow blotches as large over as a half-dollar and raised up with a yellow crust, so that I was obliged to stay away from the office a few days, when they would subside, only to recur upon my return to the office. In August I went to the country with my face unpresentable and my hands in bandages, the skin more than half gone from the affected fingers, and the nail of the index finger of the left hand in a bed of pus, so that it came off altogether. During all this time I treated my hands, I think, with all the ointments known to science for skin troubles, had a Wasserman test, urinalysis, etc., and all negative. After remaining away for two months I returned entirely well and healed. In two weeks my hands became bad again and my face so badly swollen that I was obliged to take to my bed. After getting better I began treatment of both hands and face with the violet ray by a skin specialist, from which I got some relief, and started to work again, only to be in bed again with a swollen face.

I had early discarded the use of every form of chemical used for the treatment of dead teeth. I had ceased to do much but a little exodontia and prosthesis. Physicians said that nothing could come from novocain. Finally I saw a dentist who had a similar trouble, and blamed novocain. I put aside my novocain and kept at work as best I could, and in two weeks was perfectly well again. In a few weeks I went back to my novocain, only to again start the trouble, which subsided upon ceasing to use it. I have since tried it out so many times that I am positive that novocain is a skin irritant to me. I tried novocain without the adrenalin for infiltration anesthesia and got the same irritation. It gets to the face by carelessly expelling the bubble from the syringe, which fills the air with a fine spray. The third and fourth fingers of the right hand come in contact with what liquid may have carelessly leakel out while injecting, and the index finger and thumb of the left hand come in contact with it while holding the lips or palpating. I have since found other dentists whose fingers were affected similarly to mine, and who are confident that novocain was the cause. I am now using rubber gloves, being careful when expelling the bubble from the syringe, and get along very well—Arthur E. Guptill, D.D.S., Fitchburg, Mass.

